# Detection of spontaneous breathing during an apnea test in a patient with suspected brain death using electrical impedance tomography: a case report

**DOI:** 10.1186/s12890-024-03283-4

**Published:** 2024-09-16

**Authors:** Rongqing Chen, András Lovas, Péter Bakos, Tamás Molnár, Fatime Hawchar, Balázs Benyó, Zhanqi Zhao, J. Geoffrey Chase, Stefan J. Rupitsch, Knut Moeller

**Affiliations:** 1 Institute of Technical Medicine, Hochschule Furtwangen, Jakob-Kienzle-Str. 17, Villingen-Schwenningen, 78054 Germany; 2 Department of Microsystems Engineering (IMTEK), Faculty of Engineering, University of Freiburg, Georges-Köhler-Allee 101, Freiburg, 79110 Germany; 3 Department of Anaesthesiology and Intensive Therapy, Kiskunhalas Semmelweis Hospital, Teaching Hospital of the University of Szeged, Dr. Monszpart László u. 1, Kiskunhalas, 6400 Hungary; 4 Department of Anesthesiology and Intensive Therapy, Csolnoky Ferenc Hospital, Kórház u. 1, Veszprém, 8200 Hungary; 5 Department of Cardiology, State Hospital for Cardiology, Gyógy tér 2, Balatonfüred, 8230 Hungary; 6 Department of Anesthesiology and Intensive Therapy, University of Szeged, Semmelweis u. 6, Szeged, 6725 Hungary; 7 Department of Anesthesiology and Intensive Therapy, Budapesti Dr. Manninger Jenő Traumatology Center, Fiumei út 17, Budapest, 1080 Hungary; 8 Department of Control Engineering and Information Technology, Faculty of Electrical Engineering and Information Technology, Budapest University of Technology and Economics, Muegyetem rkp. 3, Budapest, 1111 Hungary; 9 Department of Mechanical Engineering, University of Canterbury, 69 Creyke Road, Christchurch, 8041 New Zealand

**Keywords:** Apnea test, Brain death, Electrical impedance tomography, Atelectasis, Monitoring, Case report

## Abstract

**Introduction:**

The apnea test (AT) is a crucial procedure in determining brain death (BD), with detection of spontaneous breathing efforts serving as a key criterion. Numerous national statutes mandate complete disconnection of the patient from the ventilator during the procedure to open the airway directly to the atmosphere. These regulations mandate visual observation as an exclusive option for detecting breathing efforts. However, reliance on visual observation alone can pose challenges in identifying subtle respiratory movements.

**Case Presentation:**

This case report presents a 55-year-old morbidly obese male patient with suspected BD due to cerebral hemorrhage undergoing an AT. The AT was performed with continuous electrical impedance tomography (EIT) monitoring. Upon detection of spontaneous breathing movements by both visual observation and EIT, the AT was aborted, and the patient was reconnected to the ventilator. EIT indicated a shift in ventilation distribution from the ventral to the dorsal regions, indicating the presence of spontaneous breathing efforts. EIT results also suggested the patient experienced a slow but transient initial recovery phase, likely due to atelectasis induced by morbid obesity, before returning to a steady state of ventilatory support.

**Conclusion:**

The findings suggest EIT could enhance the sensitivity and accuracy of detecting spontaneous breathing efforts, providing additional insights into the respiratory status of patients during the AT.

## Introduction

The apnea test (AT) is a crucial diagnostic procedure in the confirmation of brain death (BD), defined as the irreversible cessation of all brain functions, including brainstem activity [1]. BD can result from various traumatic or medical causes, leading to brain anoxia through mechanisms, such as severe hypoxemia, impaired perfusion, or toxic injury to neurons [2]. The AT involves temporarily disconnecting the patient from mechanical ventilation, allowing the partial pressure of carbon dioxide (PaCO$_2$) to rise, lowering the cerebrospinal fluid pH to a level maximally stimulating the medullary respiratory centers [2–4]. If this center’s function is irreversibly lost, the patient will not initiate spontaneous breaths, even under adequate stimulation. Although the exact PaCO$_2$ threshold for maximal stimulation is unknown, a value of 60 mm Hg is generally accepted [5–7]. However, regulation and implementation of AT varies by country. In Hungary, national regulations require a complete disconnection of the patient from the ventilatory circuit during the test, with oxygen administered through a cannula placed in the endotracheal tube, while closely monitoring for any spontaneous respiratory effort. The apnea period typically lasts 8-10 minutes or longer if PaCO$_2$ levels do not reach the target threshold [5, 8].

A crucial aspect of the AT is the detection of spontaneous breathing efforts. The corresponding volume amplitude could be minimal and hard to observe. The test relies heavily on visual observation of the chest and abdomen by diaphragm for any movement, requiring an unobstructed view and scrutiny [2], especially in cases where regulation enables complete dismounting of the respiratory cycle. A positive AT, indicative of brain death, is characterized by the absence of any respiratory effort in response to elevated PaCO$_2$ levels. Conversely, any spontaneous respiratory activity, however subtle, constitutes a negative test, suggesting residual brainstem function [7].

Given these challenges, especially in detecting minimal respiratory efforts in cases where solely visual observation is applicable, there is a need for enhanced monitoring techniques which do not require an intact ventilatory circuit. Electrical Impedance Tomography (EIT) is a non-invasive imaging modality providing real-time monitoring of ventilation distribution and changes in lung volumes, which has been increasingly utilized in critical care settings [9]. EIT has the potential to increase the sensitivity of detecting subtle respiratory movements and provide detailed information about patient-specific lung status during the AT.

## Case

A 55-year-old male with extreme obesity (height: 170 cm, weight: 150 kg, BMI: 51.9 kg/m$^2$) was admitted to the hospital with an intracranial hemorrhage and subsequently progressed to BD. His medical history included hypertension, congestive heart failure, atrial fibrillation, diabetes, and ischaemic heart disease. Following the admission, computed tomography (CT) of the head was performed. A control scan was conducted on the second day of admission due to clinical progression. The patient was comatose, scoring Eye: 1, Verbal: tube (T), Motor: 1 on the Glasgow Coma Scale. Clinical examination revealed dilated pupils unresponsive to light, absent corneal and eyelash reflexes, and no response to pain stimuli on the trigeminal trigger points. Both vestibulo-ocular and cough reflexes were also absent. Because of the suspected BD, the first AT was scheduled on the third day of admission. Just before the test, the patient’s heart rate (HR) was 83/min. The diastolic blood pressure (DBP) was 55 mm Hg, and the systolic blood pressure (SBP) was 95 mm Hg. The mean arterial pressure (MAP) was 69 mm Hg. His body temperature was 38.1 $^\circ$C. He was receiving Isolyte^®^ infusion with a rate of 60 m*L*/h intravenously (IV), and norepinephrine with a rate of 0.9 mcg/kg/min IV.

The AT was administered under the supervision of an independent physician at the University Hospital of Szeged, Hungary, following the established protocol for determining brain death. This clinical trial is registered under NCT04857242 on ClinicalTrials.gov. The physician held the licensure required by Hungarian law for conducting such evaluations and adhered strictly to the standardized procedures regulated by the national act. Mechanical ventilation was maintained with a respiratory rate of 14/min and a pressure control setting of 24 cmH$_2$O to achieve PaCO$_2$ levels of 38-42 mm Hg, in accordance with the national Directive of the Ministry of Health on Brain Death Determination. The positive end-expiratory pressure (PEEP) was set at 10 cmH$_2$O at the discretion of the attending physician, taking into account a multi-modal monitoring strategy that optimized intracranial pressure, cerebral perfusion pressure, SaO$_2$, and PaO$_2$. With these settings and a fraction of inspired oxygen (FiO$_2$) of 0.5, arterial blood gas (ABG) analysis indicated a pH of 7.41, PaCO$_2$ of 40.5 mm Hg, and PaO$_2$ of 145 mm Hg. The patient was preoxygenated with FiO$_2$ 1.0 for 10 minutes before the apnea test. In line with Hungarian regulations, oxygen was administered during the AT at a flow rate of 6 *L*/min via a cannula inserted into the endotracheal tube, without extending beyond its tip. Electrical impedance tomography (EIT) measurements were conducted using an electrode belt (Dräger AG, Lübeck, Germany) positioned at the 5th intercostal space, with data recorded by the PulmoVista^®^ 500 (Dräger AG, Lübeck, Germany) at a frame rate of 50 Hz as part of the clinical trial.

Approximately 4 minutes following the initiation of AT, spontaneous respiratory movements were visually observed by the attending physician, leading to an immediate abortion of the AT. The patient was reconnected to the ventilator with the same settings as prior to the AT, including a PEEP of 10 cmH$_2$O and FiO$_2$ of 0.5. A slow and gradual recovery phase was observed, likely due to atelectasis induced by the open airway and further exacerbated by the patient’s morbid obesity. Following this phase, the patient’s mechanical ventilation status stabilized without further changes to the ventilator settings. ABG performed after ventilator reconnection showed a pH of 7.47, PaCO$_2$ of 35.7 mm Hg, and PaO$_2$ of 136.0 mm Hg.

In this study, the EIT signals were processed using a low-pass filter with a cutoff frequency of 70/min to exclude cardiac-related variations. To exclude potential artifacts and impedance changes from non-lung tissue and ventricular regions, the lung region was defined through linear regression analysis [10]. EIT image reconstruction was conducted using the Dräger EIT Data Analysis Tool 6.3 (Dräger AG, Lübeck, Germany). Signal filtering, lung region selection, and other quantitative analyses were performed using Matlab 2023b (Mathworks, Natick, MA, USA).

**Fig. 1:**
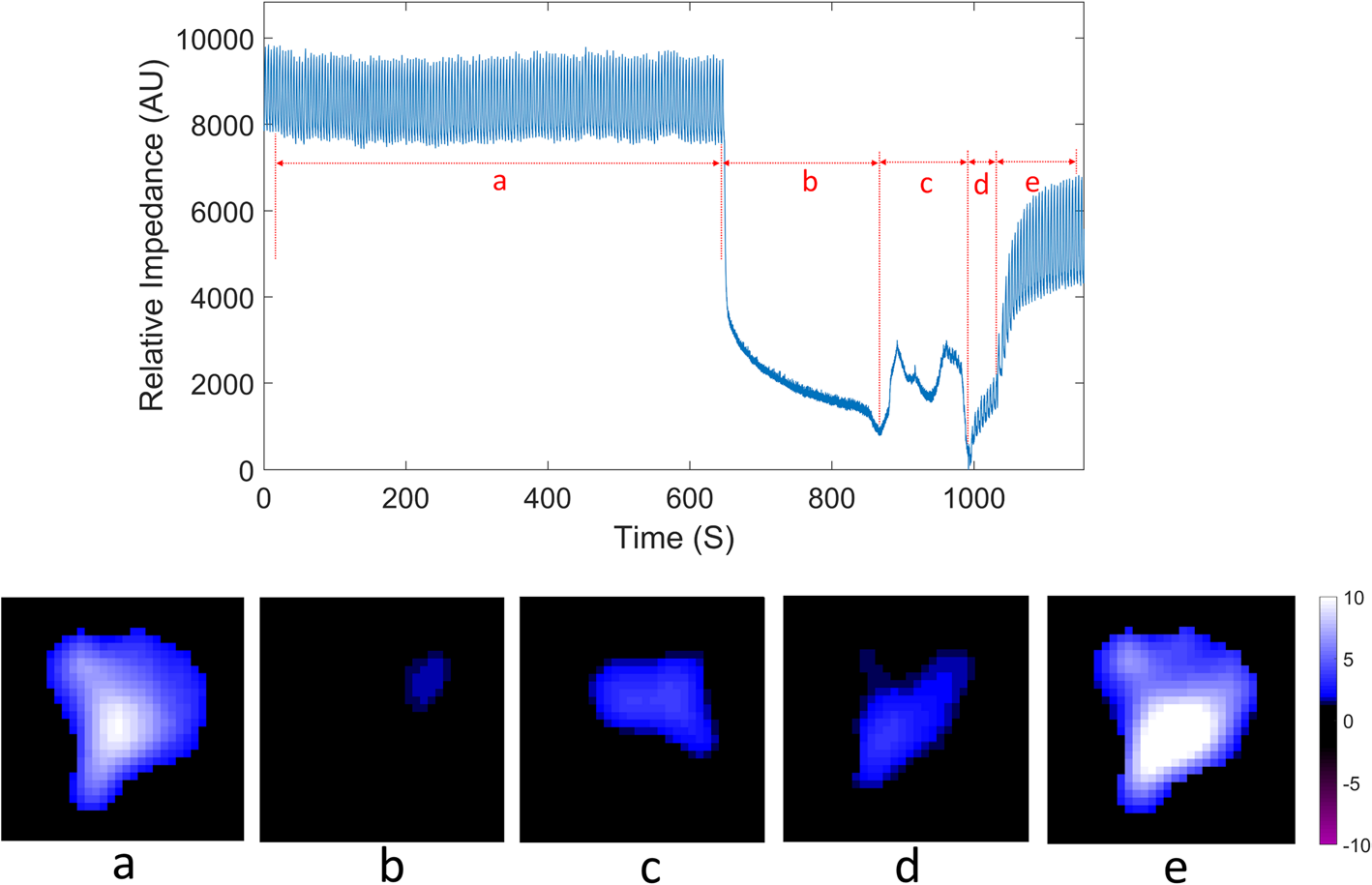
EIT monitoring results for the patient, before, during and after the apnea test. The upper row: the global impedance waveform with five signal sections; the lower row: average tidal images for each signal section: preoxygenation before the apnea test (**a**), apnea test (**b**), spontaneous breathing detected (**c**), initial recovery phase post-ventilator reconnection (**d**), stabilization at new equilibrium in mechanical ventilation (**e**). AU attributive units

The EIT monitoring results for the patient, including before the AT, during the AT, and after its abortion, are shown in Fig. 1. The upper row of Fig. 1 depicts the global impedance waveform, reflecting controlled mechanical and spontaneous ventilation. The monitoring period was divided into five signal sections: preoxygenation before the apnea test (a), apnea test (b), spontaneous breathing detected (c), initial recovery phase post-ventilator reconnection (d), stabilization at new equilibrium in mechanical ventilation (e). The EIT results depict relative conductivity changes in the lung region which do not have a unit but are strongly correlated with the tidal variations [11–13]. Usually, attributive units (AU) is used to quantify the change.

The lower row of Fig. 1 presents averaged tidal images, illustrating the tidal variation between the end-inspiratory and end-expiratory EIT images on a pixel-wise basis. During the AT (signal section b), despite the absence of mechanical ventilation support, changes in conductivity were still observed.

Tidal images during the AT were calculated as the difference image between the maximum and minimum of the global impedance waveform within signal section b. The averaged tidal images in Fig. 1 represent the mean tidal variation for each selected signal section, displayed using a consistent colormap. The tidal images during the AT show tidal variation was predominantly in the ventral part of the lungs, likely due to the oxygen flow through the endotracheal tube. Upon detecting spontaneous breathing effort, a larger tidal variation was observed, still primarily in the ventral lung regions. When mechanical ventilation was resumed, tidal variation extended to the dorsal parts of the lungs. The largest tidal variation was recorded when the patient was back to baseline mechanical ventilation after the AT (Fig. 1e).

**Fig. 2:**
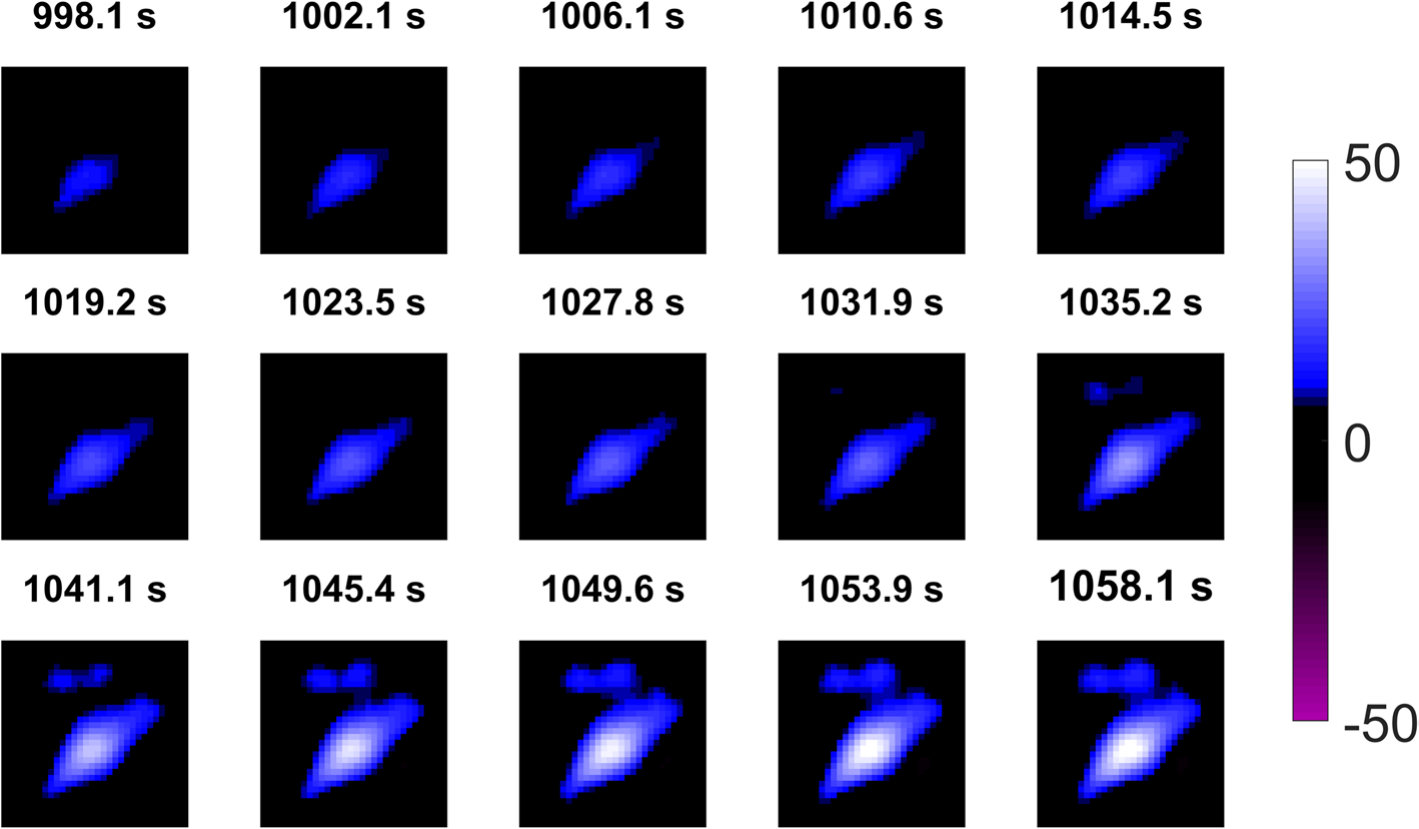
EIT images showing the gradual increase in lung aeration at the end-inspiration of each breathing cycle during the initial recovery (d) and 60 seconds into stabilization (e)

The initial recovery phase (d) is detailed in Fig. 2 regarding lung aeration. Figure 2 illustrates the EIT results depicting the aeration of the patient’s lungs at each end-inspiration, from the first end-inspiration in (d) through to 60 seconds into (e). The EIT images in Fig. 2 reveal a progressive increase in air content across both the dorsal and ventral lung regions during this period.

**Fig. 3:**
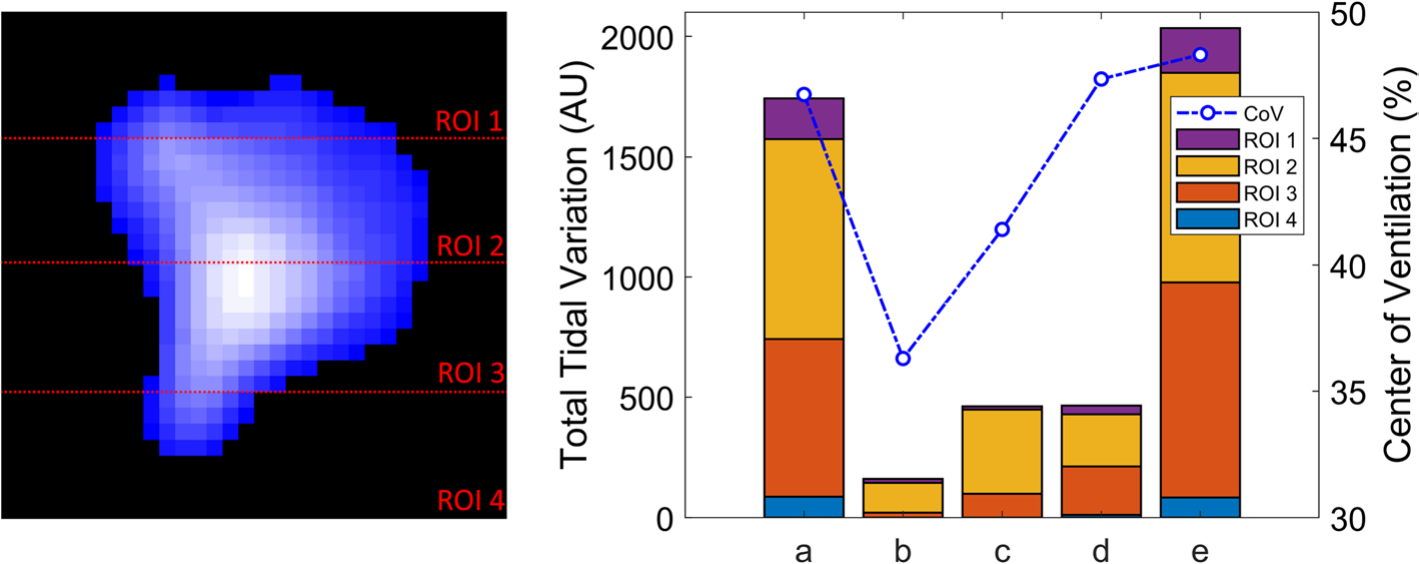
Regions of interest (ROIs) division and tidal variation across the four ROIs and center of ventilation (CoV) variation. AU attributive units

The average tidal images for each section were divided into four equal horizontal regions of interest (ROI), as depicted in the left column of Fig. 3. The right column of the figure presents stacked bar charts illustrating tidal variation across these four ROIs. During the apnea test (b) and spontaneous breathing (c), the results show tidal variation occurred mostly in ROI 2 (ventral lungs), with no significant variation in ROI 4 (dorsal lungs). Notably, the average total tidal variation during spontaneous breathing (c) and the initial recovery period after reconnecting the ventilator (d) were similar.

The center of ventilation (CoV), which measures the anteroposterior distribution of ventilation [14–16], was also calculated for each signal section and illustrated in Fig. 3. The CoV decreased during the apnea test (b), indicating a shift of ventilation distribution toward the ventral lungs. Upon the detection of spontaneous breathing (c), the ventilation distribution began shifting towards the dorsal lungs, though the CoV remained lower than during periods with mechanical ventilation support.

## Discussion

In this case report, we investigate the potential use of EIT as an additional tool for detecting spontaneous breathing during the AT in BD patients, supplementing traditional visual observation in a situation where complete disconnection of the patient was required by the regulation. Our findings indicate tidal variation during spontaneous breathing, as captured by EIT, was significantly greater than that observed during the apnea test.

Additionally, EIT revealed a shift in ventilation distribution during spontaneous breathing, which is consistent with previous observations [17], and indirectly supports the presence of a spontaneous breathing effort. Consequently, through the inhalational phase the dorsal part of the diaphragm contracts more profoundly than the ventral part [18]. Lung de-recruitment observed at the initiation of the AT was partially mitigated by the spontaneous breathing movements.

Following reconnection to the ventilator, a slow, transient, and gradual recovery was observed, followed by stabilization at a new level of lung aeration. This initial recovery could be attributed to the patient’s extreme obesity. Morbidly obese patients are prone to severe atelectasis following anesthesia with mechanical ventilation in a supine position [19]. In this case, atelectasis formation was likely more pronounced due to the absence of ventilation and an open airway to atmospheric pressure. The PEEP level of 10 cmH$_2$O was just partially able to reconvert the initial aeration of the lung. However, the calculated PaO$_2$/FiO$_2$ over 270 just after the failed AT suggested a reasonable lung opening. Increasing the PEEP further was disapproved due to the risk of a further increase in intracranial pressure.

During the AT, intentional induction of hypercarbia and respiratory acidosis aims to stimulate the medullary respiratory centers. However, the process can predispose patients to cardiopulmonary complications, such as various types of arrhythmias, fluctuation in blood pressure, or developing hypoxia [2, 20, 21]. Several modifications to the procedure have been explored to enhance safety and reduce complication rates, such as the development of severe atelectasis. These modifications include adjustments of a CPAP system, or enabling flow and capnography monitoring [5, 22–24].

However, as mentioned previously, implementation of AT highly varies according to national regulations. Despite these advancements to improve AT, the detection of spontaneous respiratory efforts continues to significantly rely heavily on visual observation of the chest and abdomen, a method whose efficacy relies on well-trained clinicians. Intensive care related overstress and inobservance due to night shift working, as well as the absence of an objective, digitally recorded and retrievable monitoring techniques, implies increased uncertainty in applying AT effectively and safely.

Our case study proposes the integration of EIT as a supplementary monitoring technique to detect spontaneous breathing effort during the AT in cases of regulation with complete disconnection from the ventilator. EIT offers the advantage of continuous and real-time imaging of ventilation distribution, offering potentially additional insights into respiratory mechanics by providing quantitative data. EIT can effectively monitor lung volume changes and detect ventilation heterogeneity in various clinical scenarios [9, 25–27]. Real-time EIT imaging provides immediate assessment of tidal impedance variations (TIV) at the bedside, which could improve the sensitivity and accuracy of the detection of subtle respiratory movements that might otherwise be missed due to distractions in visual observation. A further advantage of EIT is the digital and retrievable recording by the device, which provides a traceable audit.

It is important to note that the patient presented with extensive bilateral cerebellar hemorrhage as evidenced by CT scans, which severely compromised brain stem function. Although the patient displayed all clinical signs of brain death, spontaneous breathing persisted, likely due to minimal residual perfusion around the respiratory centers in the ventrolateral medulla oblongata, but not elsewhere. This case calls attention to the importance of AT. A series of physical examinations explored further spontaneous breath movements with the complete loss of the rest of the brain stem reflexes in the next two days. As the control CT scan revealed central nervous system damage incompatible with life and no further neurosurgical intervention emerged, patient underwent end-of-life care following the detailed information of the next of kin.

However, this case study and its implications have limitations. Integrating EIT into routine clinical practice requires the establishment of standardized protocols and comprehensive training for clinicians to ensure its effective and accurate use. Additionally, the availability and cost of EIT devices could limit the implementation of this monitoring technique. While EIT has demonstrated utility in other areas of critical care, its specific application to the AT in suspected BD determination needs to be explored further to assess its practicality and effectiveness in detecting spontaneous respiratory efforts with higher sensitivity. In addition, we cannot determine whether visual observation or EIT monitoring is more sensitive. To address this issue in future cases, we have established a protocol to input an event marker into the EIT monitoring device immediately upon the attending physician’s visual detection of spontaneous breathing. This will enable a real-time comparison of the sensitivity between visual observation and EIT monitoring.

Despite these limitations, this preliminary case study suggests that EIT could be a valuable supplementary tool in detecting spontaneous respiratory efforts during the AT. Further large-scale studies are required to validate these findings and to address the practical aspects of incorporating EIT into the clinical workflow for monitoring BD patients during the AT.

## Data Availability

The data that support the findings of this study are not openly available due to reasons of sensitivity and are available from the corresponding author upon reasonable request. Data are located in controlled access data storage at Hochschule Furtwangen.
